# An Innovative Device Based on Human-Machine Interface (HMI) for Powered Wheelchair Control for Neurodegenerative Disease: A Proof-of-Concept

**DOI:** 10.3390/s24154774

**Published:** 2024-07-23

**Authors:** Arrigo Palumbo, Nicola Ielpo, Barbara Calabrese, Remo Garropoli, Vera Gramigna, Antonio Ammendolia, Nicola Marotta

**Affiliations:** 1 Department of Medical and Surgical Sciences, Magna Graecia University of Catanzaro, 88100 Catanzaro, Italy; 2 Istituto Tecnico Industriale Statale “Enrico Fermi”, Via Piero Della Francesca, 87012 Castrovillari, Italy; 3 Garropoli Computer Science Consulting, 87100 Cosenza, Italy; remo@garropoli.it; 4 Physical Medicine and Rehabilitation Unit, Department of Medical and Surgical Sciences, University Hospital “Mater Domini”, University of Catanzaro Magna Graecia, Via Campanella, 88100 Catanzaro, Italy; ammendolia@unicz.it; 5 Research Center on Musculoskeletal Health, MusculoSkeletalHealth@UMG, University of Catanzaro “Magna Graecia”, 88100 Catanzaro, Italy; nicola.marotta@unicz.it; 6 Department of Experimental and Clinical Medicine, Magna Graecia University of Catanzaro, 88100 Catanzaro, Italy

**Keywords:** augmented reality, mixed reality, HoloLens, head-mounted display, telemedicine, remote control, smart wheelchairs, user interface, virtual reality

## Abstract

In the global context, advancements in technology and science have rendered virtual, augmented, and mixed-reality technologies capable of transforming clinical care and medical environments by offering enhanced features and improved healthcare services. This paper aims to present a mixed reality-based system to control a robotic wheelchair for people with limited mobility. The test group comprised 11 healthy subjects (six male, five female, mean age 35.2 ± 11.7 years). A novel platform that integrates a smart wheelchair and an eye-tracking-enabled head-mounted display was proposed to reduce the cognitive requirements needed for wheelchair movement and control. The approach’s effectiveness was demonstrated by evaluating our system in realistic scenarios. The demonstration of the proposed AR head-mounted display user interface for controlling a smart wheelchair and the results provided in this paper could highlight the potential of the HoloLens 2-based innovative solutions and bring focus to emerging research topics, such as remote control, cognitive rehabilitation, the implementation of patient autonomy with severe disabilities, and telemedicine.

## 1. Introduction

The World Health Organization reported that by 2021, roughly 1.3 billion individuals, constituting approximately 16% of the world’s population, were living with disabilities [1]. Moreover, over 2.5 billion people require one or more assistive products, including wheelchairs, hearing aids, or communication and cognition-supporting apps [1]. Wheelchairs stand out as one of the most effective solutions for enhancing mobility and fostering individua’ autonomy.

Several types of interfaces have been introduced so far to enable easier control of wheelchairs [2]. The most traditional method used is a joystick with a set of learned and unnatural commands to interact with the wheelchair.

Other approaches including electromyography (EMG) [3], electroencephalogram (EEG) [4,5], and/or electrooculogram (EOG) [3] signals have also been researched.

In recent years, there has been significant interest within the scientific community in utilizing the human brain for wheelchair movement and control, owing to its adaptability and potential to enhance the independence and quality of life for the elderly and people affected by neurological disorders [5,6]. Nevertheless, there has been a notable challenge associated with a high mental workload for users, particularly those with disabilities, who must constantly manage wheelchair navigation. Despite the enormous interest in implementing a brain-controlled wheelchair, the current solutions do not seem to fully satisfy the demands in today’s context, mainly due to the complexity of developing such an elaborate system [5].

An efficient opportunity to integrate people with disabilities into their everyday lives and work can be provided by new information and communication technologies (NICT) such as eXtended Reality (XR). XR encapsulates several computer-altered reality solutions that cover virtual reality (VR), augmented reality (AR), and mixed reality (MR).

By utilizing such intelligent devices, individuals with disabilities can access real-time information regarding the accessibility of buildings and locations via mobile applications. Additionally, through engagement with augmented environments integrated into the physical world, users gain enhanced visibility into building details and greater control over their environment. State-of-the-art analyses have reported only a small number of studies dealing with the wheelchair user’s requirement using VR and AR [7,8,9].

The scientific literature has shown the key significance of innovative features for wheelchair motion and command to support people in achieving independence and potentially enhancing their quality of life. In this scenario, individuals with severe cognitive, motor, or sensory impairments often use a Powered Wheelchair (PW) to satisfy their mobility demands; since they cannot handle conventional navigation methods such as classic joysticks, they might take advantage of alternative control approaches such as head joysticks, chin joysticks, sip-and-puff devices, and thought control.

In this paper, we propose a novel platform that integrates a smart wheelchair and an eye-tracking-enabled MR head-mounted display. This system is designed to allow users with severe disabilities to control the movement of a motorized wheelchair, reducing the cognitive requirements.

## 2. Background

Wheelchair movement and control are attracting widespread attention in the scientific community due to their potential to help old and paralyzed individuals gain independence and potentially improve their quality of life [2]. People with cognitive/motor/sensory impairments rely on PWs for their mobility needs, and since they cannot use the traditional solutions (classic joystick) to navigate them, they use alternative control systems (head joysticks, chin joysticks, sip-and-puff, and thought control). To meet the needs of people with disabilities who have difficulties in using power wheelchairs with daily maneuvering tasks, several researchers have proposed smart wheelchairs (SWs), employing technologies originally developed for mobile robots [10,11,12,13,14,15]. Leaman et al. [16] provided a complete state-of-the-art overview of SW research trends, classifying all available inputs methods in nine groups: Biometrics, Brain Computer Interface, Cloud, Computer Vision, Game Controller, Haptic Feedback, Multimodal, Touch, and Voice. In general, people with minor disabilities can efficiently use the wheelchair that operates using different input tools like gesture and voice [17]. Individuals with severe impairments, who need to perform multitasking using a single wearable device, are best served by augmented reality technology wheelchair aid design. Head-mounted displays (HMDs) and the concept of immersive reality related to them, are rapidly spreading in various clinical and rehabilitation sectors [17,18,19,20,21], as well as in education and training. Augmented and mixed reality have also emerged as a new way to enhance human-robot interaction (HRI) and robotic interfaces (e.g., actuated and shape-changing interfaces) and, more importantly, to help users in navigational assistance on powered wheelchairs. Although virtual reality HMDs have recently garnered attention as apt training simulators for off-line learning of wheelchair control [22,23,24], those based on augmented reality could potentially serve as a more transparent mode of communicating assistance and for on-line operation [7,8,9].

More specifically, Sawssen et al. 2019 [7] proposed a decision support system using AR (by using ORA-2 (http://www.optinvent.com/our_products/ora-2/, accessed on 1 July 2024) smart glass) for motor disabled people’s navigation assistance. The authors described a real-time wheelchair navigation system equipped with geological mapping. This solution was able to indicate access paths to a desired location and the shortest route towards it, and it identifies obstacles to avoid.

Zolotas et al. 2018 [8] proposed a novel augmented reality system using a Microsoft HoloLens 1 (Microsoft Corp., Redmond, WA, USA) as a head-mounted aid for wheelchair navigation (https://learn.microsoft.com/en-us/hololens/hololens1-hardware, accessed on 1 July 2024). The authors conducted a pilot study to investigate the influence of different interface design options. In a later study, the same authors [9] extended their earlier AR-HMD wheelchair platform [8] by adopting a new shared control and AR system, as well as by conducting a user study tailored to model misalignment. Chacón et al. [25] presented a novel augmented reality head mounted display (HoloLens 1) user interface for controlling a robotic wheelchair for people with limited mobility. To demonstrate the effectiveness of the approach, the authors evaluated their platform in two realistic scenarios: door detection and people detection.

## 3. Materials and Methods

### 3.1. Participants

The study was conducted at the rehabilitation unit of the Renato Dulbecco University hospital in Catanzaro. The test group consisted of 11 healthy subjects (six male, five female, mean age 35.2 ± 11.7 years).

The inclusion and exclusion criteria were defined by an expert medical staff. Specifically, the inclusion criteria were: 1. adult able-bodied subjects; 2. ability to identify obstacles and avoid collisions, judge speed, and distance and react quickly; 3. age greater than or equal to 18 years; and 4. signature of informed consent.

The exclusion criteria were: 1. cognitive deficit (Mini-Mental State Examination score < 24); 2. psychosis and personality disorders; 3. visual and hearing deficits; 4. severe functional limitation of the cervical spine; 5. easy muscular fatigue; 6. epilepsy in the last 24 months; 7. dependence on alcohol, narcotics, and/or psychotropic substances; and 8. cardiovascular diseases that compromise safety and driving safety.

### 3.2. The Prototype System Architecture

**Figure 1:**
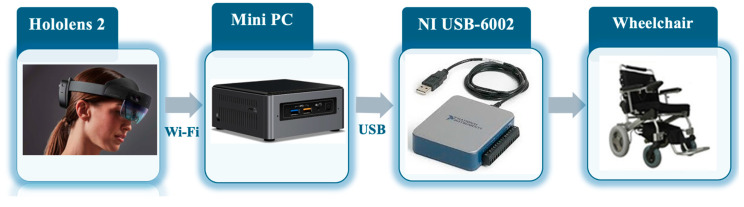
The prototype system architecture.

An ad hoc electronic circuitry was designed and placed as a mutually exclusive command to the pre-existing joystick, which can be appropriately activated via a bistable switching system (relay), which is further controlled via software. The proposed system allowed the control of the motors and therefore the movement of the wheelchair using a HoloLens 2 device (Microsoft Corp., Redmond, WA, USA). The prototype system architecture is illustrated in Figure 1.

The constitutive elements of the system are an untethered mixed reality headset, a mini-PC, the NI USB-6002 DAQ board, and the wheelchair. The technical characteristics of each of the electronic devices used are listed below.

*HoloLens 2*

**Figure 2:**
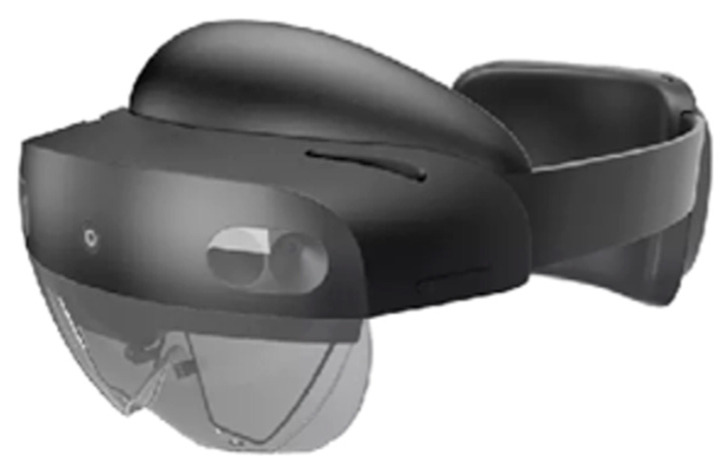
HoloLens 2 device.

As an eye-tracking enabled head-mounted display, the Microsoft^®^ HoloLens 2 ((Microsoft Corp., Redmond, WA, USA) (https://www.microsoft.com/it-it/hololens, accessed on 1 July 2024)) was considered (Figure 2). It is a novel MR-based head-mounted display (HMD) that represents a completely independent holographic computer that allows the user to interact with digital content and holograms displayed in the world around the wearer [26].

Unity 3D (v. 2021.1.28f1) and Visual Studio were used to develop the app for HoloLens 2. The management of augmented reality gives the system additional functional aspects, always aimed at enhancing the technological aids necessary to guarantee a better quality of life; in fact, the system also uses holograms to allow communication via a vocal synthesizer, which is activated by tracking eye movements directed towards a virtual keyboard.

*MiniPC*

The augmented reality device was connected via WIFI technology to a Mini PC housed in a compartment behind the wheelchair. An appropriate software has been developed using the LabVIEW development environment to allow the acquisition, decoding, and translation of visual inputs (gaze position coordinates) acquired via HoloLens 2 into control signals of the wheelchair motors.

*NI USB-6002 DAQ Board*

The actuator signals are entirely managed using the NI USB-6002 DAQ board (National Instruments, Austin, TX, USA (https://www.ni.com/it-it/shop/model/usb-6002.html, accessed on 1 July 2024)); in particular, the voltage signals necessary for controlling the two motors of the wheelchair must be included within the range from +1.2 V up to 3.8 V for both the forward and backward movement together with the left and right directions. Using the NI USB-6002 device, the control signals were appropriately modulated and sent to the electronic control unit of the motors of the motorized wheelchair.

*Wheelchair*

A commercial motorized wheelchair (a vehicle weight < 30 kg, model ET-12F22, Golden Motor Technology Co., Ltd., Changzhou, Jiangsu, CN 213164, China) was used in this study. A custom electronic board for the control of the wheelchair motors has been designed and built in advance to allow the power supply of the two wheelchair motors to be varied by means of appropriate voltage signals coming from a special electronic board of a waveform generator.

**Figure 3:**
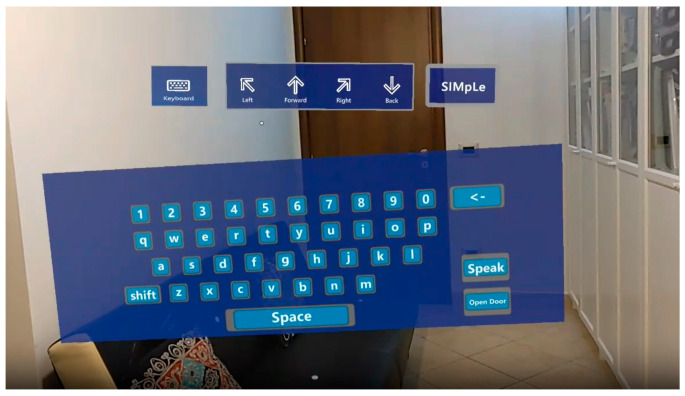
Using the virtual keyboard via Eye-Tracking (view from the HoloLens 2).

The developed system provides for the display of a hologram present at the top of the scene visible to the user, through which the user himself can make choices, simply by directing his gaze to the portion of interest of the displayed hologram. The latter consists of four graphic icons depicting four directional buttons (forward, backwards, left, right), as shown in Figure 3; the user can choose the appropriate direction by directing his gaze to the appropriate directional button, deciding moreover to block the motion of the wheelchair simply by looking away from the control hologram. The user’s choice will then be sent to the motorized wheelchair control software via a client-server socket connection.

The motorized wheelchair control software will then send to the wheelchair motor controller the appropriate voltage values to move the wheelchair forward, backward, left, and right. Therefore, the driving of the wheelchair will be managed through the movement of the user’s eyes towards the buttons of the scene, as captured by the viewer. More specifically, within the Unity3D-MKRT (Mixed Reality Toolkit) development environment of HoloLens 2, it is possible to intercept the position in which the gaze is concentrated. Using a specific function, every second, a message with the spatial coordinates of the gaze will be sent to the software wheelchair management.

A further function based on the use of a hologram depicting a keyboard (QWERTY) was also developed to allow communication with the outside world, always using eye tracking. In the main scene of the proposed application (Figure 3), there is also a button called “Keyboard”; in particular, if the user’s gaze remains fixed for at least 2 s on the keyboard icon, the keyboard will then appear in the foreground and the user can start selecting the keyboard characters by looking.

To provide further aid to communication, the system allows you to activate a “TextToSpeech” system based on a speech synthesizer capable of giving voice to what is written by the user using the keyboard; this is possible for the user by directing his gaze to the speak key on the keyboard.

### 3.3. The Testing Protocol

**Figure 4:**
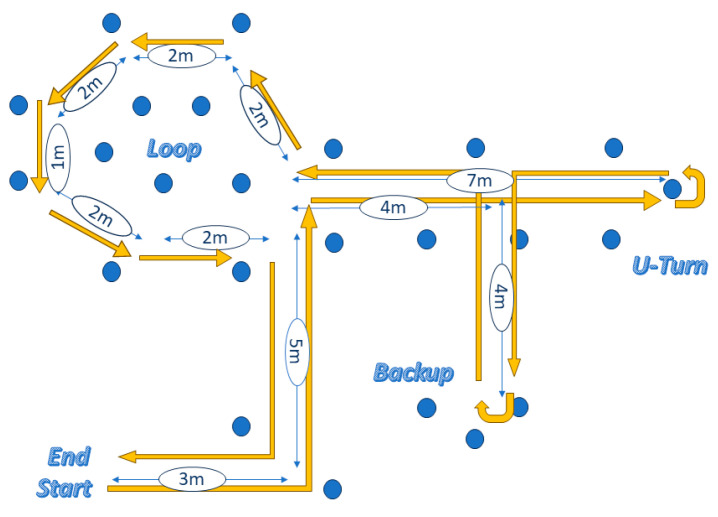
Yousefi et al. [10] circuit.

The prototype of the electronic system was tested by healthy subjects who experienced the ability to ride in a ~50 m obstacle circuit that had one loop, one backup, six turns, and twenty four obstacles, provided by Yousefi et al. [10], as depicted in Figure 4.

**Figure 5:**
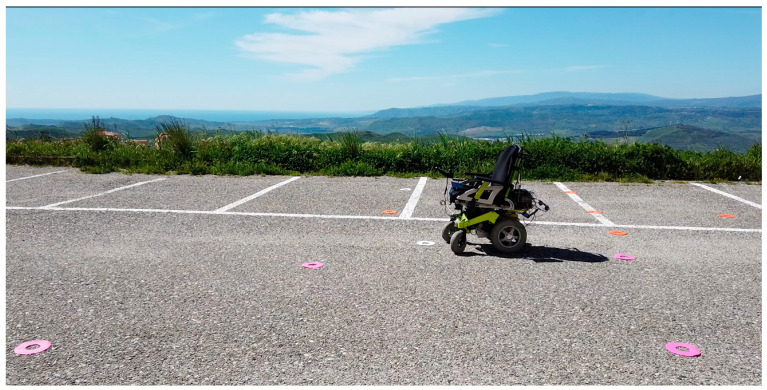
Test outdoor course for the training.

The participant drove the wheelchair three times in an analogue manner using the device’s proprietary joystick; the same participant carried out the test three more times using the HoloLens 2 device and moving the motorized wheelchair, as depicted in Figure 5.

Pre-test warm-ups were conducted with the aim to carry out the functional tests of the electric wheelchair control system with minimal effort from the user, promoting self-esteem, independence, and learning of compensatory strategies for the exploitation of residual abilities.

### 3.4. Outcome Measures

The primary outcome is to evaluate the degree of difference in speed with or without the aid of the HoloLens 2 device; however, it also includes the evaluation of autonomy and independence in movement and therefore its role in the quality of life thanks to the use of a wheelchair electric vehicle equipped with augmented reality technology.

To demonstrate the efficiency and safety of this new platform that integrates an intelligent wheelchair and an eye-tracking system, the following rating scale was used: Wheelchair Skill Test Italian version 4.2 Powered Wheelchair.

The WST also provides a total skill confidence score (0–100%) that reflects the number of skills addressed confidently (a higher score indicates greater confidence), regardless of whether the skill is mastered or not. This is of considerable importance, as training also involves learning to recognize risks and limitations.

### 3.5. Statistical Analysis

The circuit time was obtained as the mean of the three tests carried out by each subject both with (PW plus HMI group) and without the HMI (PW alone). The data were described and tabulated using R (version 3.6.0). The Shapiro–Wilk test was used to determine the normality of the data. The continuous data were presented with mean and standard deviation, while frequencies were described with percentage data. The mean differences between groups were determined using the independent t-test for data with normal distribution and the Mann–Whiney test for data with non-normal distribution. The effect sizes were presented through Cohen d (95% Confidence interval); all outcome data were calculated for within group and between group differences from different time points. The effect sizes were interpreted as minor (<0.5), adequate (between 0.5 and 0.8), and large (>0.8). For each test, the statistical analyses were two-tailed, and a *p*-value cut-off set at <0.05 was considered significant.

## 4. Results

We measured the time traveled on the circuit with or without the use of the HMI and we also presented the perceived values of WST, as depicted in Table 1.

In light of the results obtained, we report a significant non-difference in timing in completing the circuit without actually reporting any test errors (such as hitting track obstacles, device crashes, or participant safety concerns). On the other hand, functionally, the participants demonstrated significant differences in immediate functionality via WST results.

We have demonstrated a reasonable consistency of the device with useful prospects for subjects who do not have residual functionality to manage a manual joystick for daily mobility.

## 5. Discussion

The scientific community has given enormous importance to innovative solutions for wheelchair movement and control with the aim of helping old and paralyzed individuals gain independence and potentially improve their quality of life [6]. Individuals with cognitive, motor, or sensory impairments depend on PWs to fulfill their mobility requirements. Since they cannot utilize traditional navigation methods like classic joysticks, they use alternative control systems such as head joysticks, chin joysticks, sip-and-puff devices, and thought control.

To accommodate those with disabilities who find maneuvering power wheelchairs challenging in everyday tasks, numerous researchers have suggested the use of smart wheelchairs (SWs), which incorporate technologies initially developed for mobile robotics [11,12,13,14,15]. Leaman et al. [16] provided a complete state-of-the-art overview of SW research trends.

In recent years, motivated by the increasing imperative to enhance healthcare safety, the adoption of eXtended Reality (XR) technologies (including virtual, augmented, and mixed reality) across the medical field, education, and training is proving to yield significant benefits.

Indeed, for those with severe impairments who require multitasking using a single wearable device, augmented reality (AR) technology offers an optimal solution in wheelchair design. Head-mounted displays (HMDs) and the immersive reality they enable are quickly gaining traction in different clinical and rehabilitation settings [18,19,20,21], as well as in educational and training environments.

Augmented and mixed reality have also become emerging technologies to enhance human-robot interaction (HRI) and robotic interfaces (like actuated and shape-changing interfaces). Importantly, they assist users with navigation on powered wheelchairs. While virtual reality HMDs have recently attracted attention as suitable training tools for offline wheelchair control learning [23,24], those based on augmented reality could potentially offer a clearer method for conveying assistance and enabling real-time operation [7,8,9].

Specifically, Sawssen et al. 2019 [7] proposed a decision support system using AR (via ORA-2 (http://www.optinvent.com/our_products/ora-2/, accessed on 1 July 2024) smart glasses) for navigation assistance for individuals with motor disabilities. This system allows a real-time wheelchair navigation system featuring geological mapping and it could indicate the best path to a desired location as well as identify obstacles to avoid along the shortest route.

Zolotas et al. 2018 [8] introduced an innovative augmented reality system utilizing Microsoft HoloLens 1 as a head-mounted navigation aid for wheelchairs (https://learn.microsoft.com/en-us/hololens/hololens1-hardware, accessed on 1 July 2024) and conducted a preliminary study to examine the impact of various interface design options. In a subsequent study, the same authors [9] expanded their AR-HMD wheelchair platform [8], incorporating a new shared control and AR system and conducting a user study tailored to address model misalignment. Chacón et al. [25] introduced an augmented reality head-mounted display (HoloLens 1) user interface for operating a robotic wheelchair for individuals with limited mobility. The authors validated their platform’s effectiveness in two practical scenarios: door detection and human detection.

The main contribution of our paper is to present a mixed reality-based system to control a robotic wheelchair for people with limited mobility. A novel platform that integrates a smart wheelchair and an eye-tracking-enabled head-mounted display was proposed to reduce the cognitive requirements needed for wheelchair movement and control. The system prototype underwent testing with healthy participants, who successfully utilized the HoloLens 2 device for communication and operated the motorized wheelchair in various scenarios. During the activities described, the need emerged to equip the wheelchair with some additional aids, such as head support, together with a wireless safety system.

Compared to the other existing solutions [8,9,25], our proposed system aimed to manage the control and movement of a robotic wheelchair using a HoloLens 2 device ((Microsoft Corp., Redmond, WA, USA) (https://www.microsoft.com/it-it/hololens, accessed on 1 July 2024)). The Microsoft HoloLens 2 stands out as the best head-mounted display headset available in the market [26]. It boasts an exquisite design crafted from top-tier materials and provides unparalleled position-tracking capabilities. Its hands-tracking functionality operates exceptionally well, while the 3D viewing experience is remarkably realistic, with minimal object motion and exceptional stability. Notably, the HoloLens 2 has undergone a rigorous validation process, setting it apart from other commercially available systems [27]. This upgraded device features improvements in both hardware, including an expanded field of view (52°), reduced weight (566 g), and extended battery life (3 h), as well as software enhancements compared to its predecessor. Our recent review [26] offers a comprehensive overview of the technical specifications of the HoloLens 2 and conducts a thorough comparison with its predecessor, the HoloLens 1. Furthermore, it delves into the cutting-edge applications of the Microsoft^®^ HoloLens 2 within medical and healthcare contexts. The evaluations conducted in two realistic settings suggest that our platform has significant potential for application across various environments, including airports, hospitals, and office buildings, among others. Additionally, it shows promise for assisting a diverse range of disabilities. As future work, this solution will also include a system for remote monitoring biosignals, such as the ECG and EMG signals of patients. The system will be designed to monitor vital physiological user parameters and biosignals in real-time to ensure high-security conditions. Furthermore, the platform will allow medical staff to monitor the patient’s health conditions at a distance.

Nonetheless, this study is not bereft of limitations. Firstly, the study involves only 11 healthy participants; a larger, more heterogeneous sample with real-world conditions will be necessary to provide broader results. Secondly, any differences related to the participant himself, i.e., small or large BMI, type of stroke, or neurodegenerative pathology he may be suffering from, were not explored in depth. Among other things, people who are blind or have low “visus”, and/or are neurodiverse may have difficulty approaching an eye tracking system. Thirdly, there is no comparison with standard assistive devices, which could strengthen the paper; despite this, the impact of eye-tracking with the electrically powered wheelchair joystick was compared. Fourthly, we did not provide data on the follow-up safety and reliability of the system; however, despite being a proof-of-principle study, the PW plus HMI guaranteed a mean of same errors as the PW alone. Fifthly, we have not explored the use on a track with greater obstacles and architectural barriers; however, despite it being flat and free of significant obstacles, we used an asphalt track to guarantee a minimum ADL environment. Lastly, there is a growing popularity of telemedicine or telerehabilitation where the physical therapist and the person receiving care meet via video call for follow-up and treatment management. At the same time, future perspectives could open up regarding reflections on virtual workplaces, i.e., new virtual environments that could improve some accessibility problems by introducing new challenges.

## 6. Conclusions

The integration of a smart wheelchair and an eye-tracking enabled head-mounted display is a good solution to reduce the cognitive requirements needed for wheelchair movement and control, thus making the system suitable for people with limited mobility.

Additional assessments are currently ongoing to showcase the advantages of various interaction methods with the augmented reality (AR) user interface among individuals with diverse motor impairments.

Given the substantial potential of this technology, this research seeks to establish the feasibility and effectiveness of utilizing HoloLens 2 in remote control scenarios. Additionally, it aims to highlight emerging research areas such as telemedicine and motor rehabilitation.

## Figures and Tables

**Table 1:** Outcome measures for study participants.

	PW	PW Plus HMI	*p*-Value	ES	95%LCL	95%UCL
Circuit time (s)	75 ± 4.1	78.1 ± 8.5	0.064	NA	NA	NA
WST	90.6 ± 3.4	84.3 ± 4.5	0.031 *	0.674	−0.226	1.544

Abbreviations: * = significance: *p* < 0.05, ES: effect size, HMI: human machine interface, LCL: lower confidence limit, NA: not applicable, PW: Powered Wheelchair, UCL: upper confidence limit, WST: wheelchair skills test.

## Data Availability

The datasets presented in this article are not readily available because the data are part of an ongoing study.
